# Reliability and Validity of an Interviewer-Administered Adaptation of the Youth Self-Report for Mental Health Screening of Vulnerable Young People in Ethiopia

**DOI:** 10.1371/journal.pone.0147267

**Published:** 2016-02-10

**Authors:** Scott Geibel, Kassahun Habtamu, Gebeyehu Mekonnen, Nrupa Jani, Lynnette Kay, Julyata Shibru, Lake Bedilu, Samuel Kalibala

**Affiliations:** 1 Population Council, Washington, DC, United States of America; 2 School of Psychology, Addis Ababa University, Addis Ababa, Ethiopia; 3 Population Council, Addis Ababa, Ethiopia; 4 Retrak, Addis Ababa, Ethiopia; 5 Department of Psychology, Bahir Dar University, Bahir Dar, Ethiopia; University of Stellenbosch, SOUTH AFRICA

## Abstract

**Objective:**

Evaluate the reliability and validity of the Youth Self-Report (YSR) as a screening tool for mental health problems among young people vulnerable to HIV in Ethiopia.

**Design:**

A cross-sectional assessment of young people currently receiving social services.

**Methods:**

Young people age 15–18 participated in a study where a translated and adapted version of the YSR was administered by trained nurses, followed by an assessment by Ethiopian psychiatrists. Internal reliability of YSR syndrome scales were assessed using Chronbach's alpha. Test-retest reliability was assessed through repeating the YSR one month later. To assess validity, analysis of the sensitivity and specificity of the YSR compared to the psychiatrist assessment was conducted.

**Results:**

Across the eight syndrome scales, the YSR best measured the diagnosis of anxiety/depression and social problems among young women, and attention problems among young men. Among individual YSR syndrome scales, internal reliability ranged from unacceptable (Chronback’s alpha = 0.11, rule-breaking behavior among young women) to good (α≥0.71, anxiety/depression among young women). Anxiety/depression scores of ≥8.5 among young women also had good sensitivity (0.833) and specificity (0.754) to predict a true diagnosis. The YSR syndrome scales for social problems among young women and attention problems among young men also had fair consistency and validity measurements. Most YSR scores had significant positive correlations between baseline and post-one month administration. Measures of reliability and validity for most other YSR syndrome scales were fair to poor.

**Conclusions:**

The adapted, personally administered, Amharic version of the YSR has sufficient reliability and validity in identifying young vulnerable women with anxiety/depression and/or social problems, and young men with attention problems; which were the most common mental health disorders observed by psychiatrists among the migrant populations in this study. Further assessment of the applicability of the YSR among vulnerable young people for less common disorders in Ethiopia is needed.

## Introduction

HIV prevalence among young people in Ethiopia age 15 to 24 is 0.3%, with young women more vulnerable to infection than young men (0.5% female, 0.1% male). Risk of infection is higher among young people living in urban settings, including Addis Ababa (1.7% female, 0.2% male) [1]. Many young people migrate to Addis Ababa from rural areas to seek employment, and many of these migrant workers are female and have little or no education [2–3]. Many young female migrate to urban areas to escape early marriage, earn very low income as domestic workers, and are at increased risk of early sexual initiation and victimization to sexual violence [4]. Some young male migrants have no stable residence, employment, or family support system—are often classified as “street boys” as a result—and are also susceptible to sexual abuse [5–7].

Living in difficult situations such as abusive domestic employment or in street environments have been found to contribute to mental health disorders among young people. Some reports have previously described linkages between social and environmental factors and sexual abuse among young people in Ethiopia, and are often associated with negative psychosocial outcomes [8–11]. The overall prevalence of depression among adults 18 and older in Ethiopia is estimated to be 9.1%, [12] with limited information on population-based prevalence of other mental health disorders.

There is mixed evidence on how HIV-related clinical outcomes are affected by mental health disorders, although global studies show good evidence that adverse mental health conditions—especially depression—reduce antiretroviral adherence [13,14]. In Ethiopia, previous studies have investigated mental health disorders in maternal and child health contexts and settings [15,16], quality of life among HIV and tuberculosis co-infected patients [17,18], and behavioral and emotional disorders among children on antiretroviral therapy [19]. These studies found associations with mental health disorders and adverse health impacts, including childhood malnutrition and tuberculosis co-infection. The impact of targeted mental health interventions on HIV-related outcomes worldwide [14] and in Ethiopia, however, remains limited.

Towards improving the evaluation of mental health interventions for young people vulnerable to HIV in Ethiopia, a necessary initial step is to develop or adapt assessment tools by which mental health status might be measured. Review of the medical literature reveals limited systematic scale development efforts in Ethiopia, and prior documented efforts have focused mainly on validation of depression scales [20,21]. There are limited or no documented efforts to validate a scale for mental health assessment among young people specifically. The aim of this study was to select and adapt such a tool for use in Ethiopia, and assess the reliability and validity among a sample of vulnerable young people receiving social services.

## Methods

### Study population

This study was conducted among vulnerable young people receiving social services in Addis Ababa by USAID’s HIVCore Project. Young women were recruited through the Population Council’s PEPFAR-supported Biruh Tesfa project, which provides support and addresses HIV risk for migrant girls who have come to Addis Ababa for work. Services provided by Biruh Tesfa include basic education, life skills training, and mental and physical health referrals. Young men were recruited through Retrak, a non-governmental organization which primarily works to empower street boys to obtain more stable social living environments, health care, shelter, and support.

To be eligible for this study, all participants were required to be registered recipients of services from Biruh Tesfa or Retrak within the past 3 months, age 15 to 18, and able to provide informed consent. While Biruh Tesfa and Retrak provide services to both older and younger people outside of the 15 to 19 age range, the minimum age of this study was 15 due to ethical and consent considerations. A maximum age of 18 was set, as the final screening tool selected for this study was designed for people age 18 or younger.

### Measures

#### Selection, background, structure, and adaptation of the Youth Self Report (YSR)

To initially identify and select a mental health screening tool for adaptation, a committee of psychiatrists, psychologists, public health professionals, service providers, and social science researchers was convened in Addis Ababa, Ethiopia. Screening tools previously validated outside of Ethiopia were reviewed and discussed, including the Symptom Assessment 45 Questionnaire [22,23], the Symptom Checklist 90 [24], the Strengths and Difficulties Questionnaire [25], and the Achenbach System of Empirical Based Assessment (ASEBA) [26]. The committee considered the applicability of these tools in Ethiopia based on the following criteria: guidance available for administration, scoring, and results interpretation; ease of administration; applicability of the tool for the target age group; and adequate coverage range of mental health conditions, including anxiety and depression. Upon review of the above, the committee selected the ASEBA Youth Self-Report (YSR) form for administration.

The YSR is a widely used self-administered questionnaire for the assessment of emotional and behavioral issues among young people age 11 to 18 years. Previous studies have established the YSR as having generally good internal consistency and test-retest reliability [26], with variable cross-informant agreement across societies [27]. Other studies have assessed the content validity of YSR scales with generally strong historical results [26], including more recent assessments assessing the YSR as a measure of depression [28], suicidality [29], and anxiety and affective problems [30,31]. The YSR has frequently been the subject of assessments of criterion validity, sometimes as a primary instrument of evaluation [32,33], but the YSR is also frequently utilized as a comparison tool in validation studies assessing newer original or adapted scales [34–41].

Geographic application of the YSR has been broad-based, including recent worldwide reports of YSR use in North America [42,43], South America [44], Europe [45,46], Asia [47–49], Australia [50], Middle East and North Africa [51,52], and sub-Saharan Africa [53]. However, studies objectively assessing the development, reliability, and validity of the YSR in developing countries are more limited [54, 55].

In this study, a total of 105 items from the YSR questionnaire were used to assess the validity and reliability of scores for eight syndrome scales (anxious/depressed, withdrawn/depressed, somatic complaints, social problems, thought problems, attention problems, rule-breaking behavior, and aggressive behavior), an internalizing problem scale (combining scores from anxious/depressed, withdrawn/depressed, and somatic complaints), and an externalizing problem scale (combining scores from rule breaking behavior and aggressive behavior). YSR items are framed as statements about a life experience within the past 6 months, whereupon respondents provide a response of “not true” (scored as 0), “somewhat or sometimes true” (1), or “very true or often true” (2). Additionally, scales designed to approximate diagnoses from the Diagnostic and Statistical Manual of Mental Disorders, 4^th^ Edition (DSM-IV) [56] were scored for affective problems, anxiety problems, somatic problems, attention deficit/hyperactivity problems, oppositional defiant problems, and conduct problems. All scales and scores were defined and calculated according to ASEBA guidelines [26,57].

Initial adaptation of phrasing and content of some YSR items, and forward translation of the YSR questionnaire from English to Amharic, was conducted by a psychologist, whereupon the Amharic version was reviewed by the study advisory committee comprised of the lead study psychologist (Bahir Dar University), a psychometrician (Addis Ababa University), an independent psychiatrist (Amanuel Mental Health Hospital), service providers from Biruh Tesfa and Retrak, and research officers from the study implementers (HIVCore/Population Council). After discussion and modification, the Amharic was then back-translated to English by a professional translator. Both Amharic and back-translated English versions were again reviewed by the study advisory committee, until the Amharic version was pilot tested, further modified, and then finalized.

#### Psychiatric assessment

After the participants completed the Amharic version of the YSR, two Ethiopian licensed junior psychiatrists trained at Addis Ababa University (blinded from the YSR results) conducted clinical assessments of participants. For this study, the psychiatrists recorded confidential examination notes, and then recorded if the respondents had a clinical diagnosis equivalent to any of the eight syndrome scales scored by the YSR (anxious/depressed, withdrawn/depressed, somatic complaints, social problems, thought problems, attention problems, rule-breaking behavior, and/or aggressive behavior). This was recorded categorically as “yes” (diagnosed) or “no” (not diagnosed). The psychiatrists were trained in accordance with international standards and in cooperation with international partners [58,59]. Study participants who were diagnosed by the psychiatrists were given appropriate referrals in accordance with the World Health Organization’s evidence-based mental health Gap Action Programme (mhGAP) guidelines for diagnosis and treatment [60]. Study procedures.

Given low expected levels of literacy among the target population, the questionnaire was modified to be administered by trained and experienced nurses. At baseline, young men receiving services from the Retrak project and young women involved in the Biruh Tesfah project were approached and invited by the study team using a convenience sampling based on the eligibility criteria. After obtaining informed consent from participants, the YSR was administered and then immediately followed by the psychiatrist assessment. Participants were then invited to repeat both assessments after a period of one month.

### Data analysis

YSR questionnaires and psychiatrist forms were entered into an electronic database, checked for accuracy, and analyzed using IBM SPSS Statistics 21.0 (Armonk, NY: IBM Corp.). Median YSR scores were computed according to guidelines in the ASEBA manual [26].

#### Handling of missing data

Given that the YSR was administrated to participants by trained counselors and clinical nurses in this study, missing data levels were minimal (0.20% of all values). No single YSR item had more than 3.0% (4/134) of case data missing, and no participant had more than 3 of 105 (2.9%) total YSR items missing. Of all participants, 17.2% (23/134)—25.4% of 67 young women and 9.0% of 67 young men—had at least one of the 105 items missing. Within the YSR subscales, however, no more than 4.5% (6/134) of participants had missing data, with the highest at 7.5% (5/67) among young women and 1.5% (1/67) among young men.

For the analysis of internal reliability, missing values were not replaced. For the analysis of test-retest reliability and criterion validity of YSR scores, to maintain optimal sample size and variability in the samples respondents were assumed to not have a “not true” response and items with missing values were given a value of 0. The analysis was repeated without replacement of missing values (not presented in this manuscript) and results were similar and yielded the same conclusions and interpretation.

#### Reliability

Internal consistency of the responses on each of the YSR scales was assessed with Cronbach’s alpha coefficients. Differences in coefficients between young women and men were tested for statistical significance [61,62].

Test-retest reliability was assessed through comparison of scores among participants who were administrated the YSR at both baseline and one month later. Scores on YSR scales were assessed for distribution, and differences between baseline and follow-up medians were tested using the Wilcoxon signed-rank test. To measure correlations between baseline and one-month follow-up scores, Pearson’s product-moment correlation coefficients were calculated.

#### Validity

Criterion validity was assessed by analyzing the concurrence between the baseline (first interview) YSR scores and the psychiatric assessments. Scores of participants who were reported by psychiatrists to have a mental disorder in line with the YSR syndrome scales were compared to scores of participants who did not. Measures of central tendency (median and mean) and the underlying distribution of the data were assessed, and the Mann-Whitney U test was used to determine significant differences. A receiver operating characteristic (ROC) analysis was conducted to determine “area under the curve” (AUC) values, which measure the degree to which the YSR scores predict the binary measurement of the psychiatric diagnosis (participant has a mental health problem, does not have a problem).

For scores disaggregated by sex (female participants in Biruh Tesfa, and male participants in Retrak) that were found to be meaningfully (*P*<0.05) or marginally (*P*<0.12) statistically associated with psychiatric assessments, the sensitivity and specificity of the selected YSR syndrome scores were evaluated.

### Ethical review

This study was reviewed and approved by the Ethiopia Ministry of Science and Technology National Research Ethics Review Committee (NRERC) and the Population Council Institutional Review Board. All study participants gave written informed consent. Participants age 15 to 17 were living in migrant situations requiring them to make serious daily life decisions on their own, and many were not living with a parent or guardian. Based on their independent living status signified by enrolment in Biruh Tesfa and Retrak projects, all participants age 15 to 17 were classified as emancipated minors, and provided written informed consent on their own behalf. The ethics committees approved the classification of the study population as emancipated minors on this basis, and fully reviewed and approved the consent procedures and forms. The data used for this study were anonymized and not linked to personal information in project or other records.

## Results

A total of 134 young people were administered the adapted YSR, including 67 young men enrolled in the Retrak project and 67 young women in Biruh Tesfa (Table 1). The mean age of both participant groups was 15.7 years. The majority of participants were of Oromo (40.3%) or Amhara (26.1%) ethnicity, with substantial differences between young men and women in Welayta (23.9 versus 3.0%) and Gurage (3.0% versus 11.9%) enrollment. Young men in the study were most likely to report earning money through daily labor activities (79.1%), while almost all the young women worked as housemaids (97%). Most of the respondents were not currently in school (data not shown).

**Table 1:** Basic characteristics of participants assessed by the YSR.

Characteristic	Young men (Retrak, n = 67)		Young women (Biruh Tesfa, n = 67)		Total (n = 134)	
	n	%	n	%	n	%
**Sex**						
Male	67	100.0	-	-	67	50.0
Female	-	-	67	100.0	67	50.0
**Age (years; mean and SD)**	15.7 (±1.0)		15.7 (±0.8)		15.7 (±0.9)	
15	34	50.7	43	64.2	77	57.5
16	21	31.6	9	13.4	30	22.4
17	11	16.4	9	13.4	20	14.9
18	1	1.5	6	9.0	7	5.2
**Ethnicity**						
Oromo	26	38.8	28	41.8	54	40.3
Amhara	13	19.4	22	32.8	35	26.1
Welayta	16	23.9	2	3.0	18	13.4
Gurage	2	3.0	8	11.9	10	7.5
Others (<5% each)	10	14.9	7	18.9	17	12.7
**Primary source of income**						
Housemaid	NR	NR	65	97.0	65	48.5
Daily labor	53	79.1	1	1.5	54	40.3
Shoe shining	4	6.0	NR	NR	4	3.0
Other	6	9.0	1	1.5	7	5.2
None	4	6.0	NR	NR	4	3.0

SD, standard deviation

### Reliability

Consistency on most individual YSR syndrome scales for both young men and women ranged from unacceptable (α<0.5) to poor (α≥0.5 and α<0.6) (Table 2). The YSR showed acceptable (α≥0.6 and α<0.7) to good (α≥0.7) internal consistency with the anxious/depressed (α = 0.628 young men; α = 0.710 young women) and withdrawn/depressed (α = 0.549 men; α = 0.624 women) scales. The YSR also showed much better internal consistency for young men than women for the rule-breaking (α = 0.57 versus α = 0.115, *P* = 0.007) and aggressive (α = 0.693 versus α = 0.409, *P* = 0.014) behavior scales. This was reflected in significantly different broad externalizing scale scores (α = 0.750 versus α = 0.428, *P* = 0.002), while internalizing scale scores showed good internal consistency for both young men (α = 0.767) and women (α = 0.812). Results were similar in the DSM-oriented scales, where internal consistency was stronger for both young men and women in the affective problems scale (α = 0.721 and α = 0.683); while differing significantly in the somatic problems (0.477 versus α = 0.000, *P* = 0.025), oppositional defiant problems (α = 0.707 versus α = 0.267, *P* = 0.003), and conduct problems (α = 0.600 versus α = 0.059, *P* = 0.002) scales.

**Table 2:** Internal consistency of the YSR among young men and women in Ethiopia.

Youth Self Report (number of items)	Young men (Retrak, n = 67)	Young women (Biruh Tesfa, n = 67)	Test of difference in coeffiecients	Total (n = 134)
	Cronbach's α	Cronbach's α	(χ2) *P*-value	Cronbach's α
**Syndrome scales**				
Anxious/depressed (13)	0.628	0.710	(0.83) 0.362	0.646
Withdrawn/depressed (8)	0.549	0.624	(0.42) 0.516	0.608
Somatic complaints (10)	0.436	0.347	(0.29) 0.593	0.385
Social problems (11)	0.465	0.523	(0.18) 0.627	0.476
Thought problems (12)	0.492	0.521	(0.05) 0.831	0.499
Attention problems (9)	0.537	0.451	(0.38) 0.540	0.540
Rule-breaking behavior (15)	0.570	0.116	**(7.31) 0.007**	0.620
Aggressive behavior (17)	0.693	0.409	**(6.04) 0.014**	0.662
**Broad band scales**				
Internalizing (31)	0.767	0.812	(0.67) 0.413	0.785
Externalizing (32)	0.750	0.428	**(10.11) 0.002**	0.755
**DSM-oriented scales**				
Affective problems (13)	0.721	0.683	(0.22) 0.638	0.703
Anxiety problems (6)	0.479	0.521	(0.80) 0.777	0.529
Somatic problems (7)	0.477	0.000	**(5.04) 0.025**	0.442
Attention deficit/hyperactivity problems (7)	0.546	0.230	(3.39) 0.066	0.487
Oppositional defiant problems (5)	0.707	0.267	**(8.88) 0.003**	0.595
Conduct problems (15)	0.600	0.059	**(10.14) 0.002**	0.622

After one month, 50 of the 67 young male participants (74.6%) and 61 of the 67 female participants (91.0%) were re-administered the YSR. Across nearly all the syndrome, broad band, and DSM-oriented scales; scores were significantly lower one month later (Table 3). The one-month scores, however, mostly remained positively and significantly correlated with the baseline YSR scores.

**Table 3:** Comparison and correlations of YSR scores between baseline and 1-month follow-up measurements.

Youth Self Report (number of items)	Young men (Retrak, n = 50)			Young women (Biruh Tesfa, n = 61)			Total (n = 111)		
	Baseline	1-month	*r*	Baseline	1-month	*r*	Baseline	1-month	*r*
	Median, IQR	Median, IQR		Median, IQR	Median, IQR		Median, IQR	Median, IQR	
**Syndrome scales**									
Anxious/depressed (13)	6, 4–9	2, 1–4**	0.31*	6, 4–9	3, 2–5)**	0.67**	6, 4–9	3, 1–4**	0.55**
Withdrawn/depressed (8)	4, 3–6	2, 1–4**	0.51**	6, 4–8	4, 3–6**	0.59**	5, 3–7	3, 1–4**	0.59**
Somatic complaints (10)	1, 0–2	0, 0–0**	0.29*	1, 0–2	0, 0–1**	0.60**	1, 0–2	0, 0–1**	0.48**
Social problems (11)	4, 2–5	2, 1–3**	0.30*	3, 1–4	1, 0–2**	0.54**	3, 2–5	1, 1–2**	0.45**
Thought problems (12)	1, 0–1	0, 0–0**	0.10	0, 0–1	0, 0–0**	0.46**	0, 0–1	0, 0–0**	0.32**
Attention problems (9)	3, 1–4	1, 0–2**	0.17	2, 1–3	1, 0–2**	0.34**	2, 1–4	1, 0–2**	0.25**
Rule-breaking behavior (15)	3, 0–1	1, 0–2**	0.53**	1, 0–1	0, 0–1	0.21	1, 0–3	1, 0–2**	0.56**
Aggressive behavior (17)	3, 1–5	1, 0–3**	0.53**	1, 0–3	0, 0–1**	0.31*	2, 1–3	0, 0–2**	0.49**
**Broad band scales**									
Internalizing (31)	10, 7–14	5, 3–8**	0.53**	13, 10–17	8, 4–10**	0.73**	12, 8–16	6, 4–9**	0.67**
Externalizing (32)	5, 3–9	3, 1–4**	0.59**	2, 1–4	1, 0–2**	0.31*	3, 2–6	1, 0–4**	0.59**
**DSM-oriented scales**									
Affective problems (13)	4, 2–6	1, 1–3**	0.35*	4, 2–5	2, 0–3**	0.72**	4, 2–6	2, 1–3**	0.53**
Anxiety problems (6)	1, 1–3	0, 0–0**	0.38**	3, 1–4	2, 1–2**	0.58**	2, 1–4	1, 0–2**	0.58**
Somatic problems (7)	0, 0–0	0, 0–0*	NA	0, 0–0	0, 0–0	-0.05	0, 0–0	0, 0–0	-0.04
Attention deficit/hyperactivity problems (7)	1, 0–3	0, 0–1**	0.39**	1, 0–2	0, 0–1**	0.41**	1, 0–2	0, 0–1**	0.38**
Oppositional defiant problems (5)	1, 0–2	0, 0–1**	0.46**	1, 0–2	0, 0–1**	0.04	1, 0–2	0, 0–1**	0.31**
Conduct problems (15)	2, 0–3	1, 0–2*	0.53**	0, 0–1	0, 0–1	0.07	1, 0–2	1, 0–1*	0.53**

SD, standard deviation; *r*, Pearson product-moment correlation. Significant differences in medians between baseline and 1-month data from the Wilcoxon signed-rank test are designated by asterisks after the 1-month measurement. Significance in correlations are designated by asterisks after the *r* statistic.

*P<0.05;

**P<0.01

### Validity

Table 4 compares the baseline YSR median syndrome scores between participants who did, and did not, receive a confirmatory psychiatric diagnosis, and reports the results of the corresponding ROC analyses. Psychiatric diagnoses were most strongly associated with higher median scores and predictive ROC curves for the anxious/depressed scale for young women (*P* = 0.003; AUC = 0.846, *P* = 0.005), the social problem scale for young women (*P* = 0.004; AUC = 0.774, *P* = 0.004), and the attention problems scale for young men (*P* = 0.100; AUC = 0.850, *P* = 0.094).

**Table 4:** Comparison of YSR syndrome scales by confirmatory psychiatric diagnosis; scoring and ROC AUC values.

			Comparison of median scores			Receiver operating characteristic (ROC) analysis		
YSR syndrome scales	Sex	Confirmatory psychiatric diagnosis?	n	Median, IQR	*P*-value	AUC	SE	*P*-value
Anxious/depressed	Women	Yes	6	11, 9–12	0.003	0.846	0.054	0.005
		No	61	6, 4–8				
	Men	Yes	6	7, 5–9	0.395	0.609	0.103	0.380
		No	61	6, 3–9				
	Total	Yes	12	9, 7–12	0.009	0.729	0.066	0.009
		No	122	6, 3–9				
Withdrawn/depressed	Women	Yes	1	10	0.179	0.924	0.034	0.148
		No	61	6, 4–8				
	Men	Yes	0	-	-	NA		
		No	67	3, 3–5				
	Total	Yes	1	10	0.104	0.951	0.022	0.121
		No	133	4, 3–7				
Somatic complaints	Women	Yes	0		-	NA		
		No	67	1, 0–2				
	Men	Yes	2	1, 0–2	0.926	0.523	0.218	0.912
		No	65	1, 0–2				
	Total	Yes	2	1, 0–2	0.919	0.477	0.217	0.912
		No	132	1, 0–2				
Social problems	Women	Yes	11	6, 3–7	0.004	0.774	0.083	0.004
		No	56	3, 1–4				
	Men	Yes	11	5, 4–6	0.116	0.649	0.094	0.119
		No	56	3, 2–5				
	Total	Yes	22	5, 4–7	0.001	0.715	0.064	0.001
		No	112	3, 2–4				
Thought problems	Women	Yes	0	-	-	NA		
		No	67	1, 0–1				
	Men	Yes	0	-	-	NA		
		No	67	1, 0–2				
	Total	Yes	0	-	-	NA		
		No	134	1, 0–2				
Attention problems	Women	Yes	0	-	-	NA		
		No	67	2, 1–3				
	Men	Yes	2	8, 4–11	0.100	0.850	0.115	0.094
		No	65	3, 1–4				
	Total	Yes	2	8, 4–11	0.050	0.894	0.080	0.056
		No	132	2, 1–4				
Rule-breaking behavior	Women	Yes	0	-	-	NA		
		No	67	1, 0–1				
	Men	Yes	2	4, 3–5	0.458	0.665	0.111	0.428
		No	65	3, 1–4				
	Total	Yes	2	4, 3–5	0.129	0.826	0.063	0.115
		No	132	1, 0–3				
Aggressive behavior	Women	Yes	0	-	-	NA		
		No	67	1, 0–3				
	Men	Yes	1	4	0.627	0.697	0.059	0.501
		No	66	3, 1–5				
	Total	Yes	1	4	0.433	0.786	0.041	0.326
		No	133	2, 1–3				

IQR, interquartile range; SD, standard deviation; AUC, area under the curve; SE, standard error; NA, not applicable due to no psychiatric diagnosis

For these three syndrome scales, Table 5 shows the predictive sensitivity and specificity of selected scores. For anxiety/depression scores, the optimal female score of ≥ 8.5 has a sensitivity of 0.833 and specificity of 0.754, while a total—or combined male and female—score of ≥ 6.5 was less predictive with 0.750 sensitivity and 0.631 specificity. The social problems score for young women of ≥ 3.5 had a sensitivity of 0.727 and specificity of 0.679, while the same score for men was more sensitive (0.818) but much less specific (0.536). Only young men were diagnosed with attention problems and the scale score of ≥ 3.5 was very accurate with a sensitivity of 1.000, but much less specific at 0.600.

**Table 5:** Sensitivity and specificity for diagnosis of anxious/depression problems, social problems, and attention and rule-breaking problems.

Problem	Score	Sensitivity	Specificity
**Anxiety/depression**			
Women	≥ 6.5	1.000	0.639
	≥ 7.5	0.833	0.689
	≥ 8.5	0.833	0.754
	≥ 9.5	0.667	0.820
Men	≥ 4.5	0.833	0.328
	≥ 5.5	0.667	0.443
	≥ 6.5	0.500	0.623
Total	≥ 5.5	0.833	0.443
	≥ 6.5	0.750	0.631
	≥ 7.5	0.667	0.689
**Social problems**			
Women	≥ 2.5	0.818	0.482
	≥ 3.5	0.727	0.679
	≥ 4.5	0.545	0.839
Men	≥ 2.5	0.818	0.304
	≥ 3.5	0.818	0.536
	≥ 4.5	0.636	0.679
Total	≥ 2.5	0.818	0.393
	≥ 3.5	0.773	0.607
	≥ 4.5	0.591	0.759
**Attention problems**			
Men	≥ 2.5	1.000	0.477
	≥ 3.5	1.000	0.600
	≥ 4.5	0.500	0.800

## Discussion

This study adapted an interviewer-administered Amharic version of the YSR, and findings indicate that the YSR has sufficient reliability and validity in identifying young vulnerable women with two of the eight syndrome scales (anxiety/depression and/or social problems), and young men with one of the syndrome scales (attention problems), in Addis Ababa, Ethiopia. This study did not measure acceptable reliability and validity for the remainder of the eight YSR syndrome scales. The YSR syndrome scale scores observed in this study for anxiety/depression and social problems reflected the most common manifestations of these problems observed among young men and women by psychiatrists within the study population. The effectiveness of the YSR for identifying depression problems, particularly, is also consistent with other studies that have tested the YSR in other settings [28,29,33].

Analysis in this study may have been affected by the smaller sample sizes, in part due to low prevalences of some of the disorders that were assessed by the YSR. Internal consistencies as measured by Cronbach’s alpha were lower than expected. While the literature conveys that alphas of ≥ 0.70 reflect ideal internal consistency [63,64,65], this study only measured α ≥ 0.70 on one individual syndrome scale (young women, anxious/depressed) and only measured α ≥ 0.60 in three cases (young women, withdrawn/depressed; and young men, anxious/depressed and aggressive behavior). The broad band scores of reflecting internalizing and externalizing items showed good internal consistency, although this is expected for most scales with larger numbers of items [66]. Reliability and validity of the YSR for measuring somatic problems among young men, and especially for defiant/aggressive problems among young girls, were poor. This is probably the result of low prevalences of these disorders in the study populations.

This study’s assessment of test-retest reliability was limited in that the one-month retest was biased by what may have been an intervention effect. A full assessment of intervention impact will be conducted under a separate study. The participants’ involvement in Retrak and Biruh Tesfa programming was ongoing during this study, which may have influenced the improvements in participant mental health status as shown in Table 3. Another limitation was that the psychiatrist assessments for this study were not based on a standardized and/or validated clinical tool and categorized the diagnoses according to the YSR domains, and thus there may have been some incorporation bias in diagnoses or syndrome classification as a result.

Despite these limitations, the YSR scores in this study highlighted plausible disorders among the young women who were mostly domestic workers (depression and social problems) and young men who were predominantly street-based laborers (attention problems). The results also correlated well with disorders observed by study psychiatrists. As a result, the study advisory committee determined that the YSR could be used in Ethiopia to indicate these gender-specific disorders. At the same time, YSR scores may differ in meaning or interpretation between young men and women, as shown by differences by sex in sensitivity and specificity between similar scores.

Results from this study and a separate needs assessment were used to inform the intervention development, of which implementation and analysis is currently ongoing. Further assessment is needed to refine and verify these results, as well as determine whether administration of the full 105-item YSR is efficient and necessary with young migrant populations, or if a brief version of the YSR may be adapted. Efforts to continue to identify the mental health needs and intervention needs for vulnerable young people in Ethiopia are encouraged.

## Data Availability Statement

Ethical and legal restrictions make data unsuitable for public deposition. Contact dataaccess@popcouncil.org to request an anonymized dataset.

We are unable to provide the YSR tool used by this study as a supplemental file due to legal restrictions. More information and a copy of the YSR questionnaire is available at www.aseba.org.
